# IL-12 stimuli during ex vivo expansion not only prevents the upregulation of PD1 on tumor infiltrating adoptively transferred T cells but also on endogenous TILs which results in effective tumor regression

**DOI:** 10.1186/2051-1426-2-S3-P10

**Published:** 2014-11-06

**Authors:** C Marcela Diaz-Montero, Yu Yang, Patricia A Rayman, Alberto Montero, Charlie Tannenbaum, James Finke

**Affiliations:** 1 Cleveland Clinic Foundation, Cleveland, OH, USA; 2 Cleveland Clinic, Cleveland, OH, USA

## 

The blockade of negative immune regulators such as CTLA-4 and PD1 continues to show encouraging results in patients with solid malignancies. The efficacy of these therapies could be further enhanced in a combinatorial setting that includes adoptive cell therapy (ACT), and thus it is important to clearly understand the relative impact that checkpoint blockade has on the anti-tumor activity of transferred and endogenous CD8^+ ^T cell populations. Low ACT efficacy is associated with increased levels of PD1 by tumor infiltrating transferred T cells; IL-12 stimulation during activation prevents the re-expression of PD1 within the tumor. Here we show that *ex vivo *activation of Pmel cells in the presence of IL-12 (Pmel^12^) not only maintains low levels of PD1 expression once they reach the tumor, but also has an impact on the expression of PD1 by endogenous tumor infiltrating lymphocytes (TILs). Low levels of PD1 expression by both tumor infiltrating Pmel^12 ^cells and TILs correlated with effective regression of primary tumors. In the periphery, however, both endogenous and transferred CD8^+ ^T cells showed low levels of PD1 expression regardless of IL-12 stimuli during *ex vivo *activation. We assessed the ability of these circulating CD8^+ ^T cell populations to prevent the dissemination of tumors following intravenous injection of B16 melanoma cells. Mice treated with either Pmel^12 ^cells or Pmel cells activated in the presence of antigen only (Pmel^Ag^) were both able to effectively prevent the spread of tumor cells to the lung. Interestingly, this occurred despite robust progression of primary tumors in mice treated with Pmel^Ag ^cells. This differential expression of PD1 between tumor infiltrating (high PD1) and circulating tumor reactive CD8^+ ^T cells (low PD1) might explain the failure to control the progression of localized tumor lesions after transfer of Pmel^Ag ^cells while effectively preventing the establishment of distant ones. These findings suggest that factors within the tumor other than antigen modulate the expression of PD1 on infiltrating T cells. Thus, expanding T cells for ACT in the presence of IL-12 represents a feasible strategy to protect tumor infiltrating CD8^+ ^T cells from tumor derived PD1-inducing factors which otherwise would hinder the efficacy of ACT.

